# Physical Activity as a Predictor of Emotional Quality of Life in Postmenopausal Women

**DOI:** 10.3390/healthcare14040466

**Published:** 2026-02-12

**Authors:** Adrianna Maria Kosior-Lara, Jacek Wąsik, Małgorzata Kuchta, Dorota Ortenburger, Agnieszka Pluto-Prądzyńska

**Affiliations:** 1 Department of Nursing, Jan Dlugosz University, 42-200 Częstochowa, Poland; a.kosior-lara@ujd.edu.pl; 2 Institute of Physical Culture Sciences, Jan Dlugosz University, 42-200 Częstochowa, Poland; j.wasik@ujd.edu.pl (J.W.); d.ortenburger@ujd.edu.pl (D.O.); 3 Institute of Technology, State University of Applied Sciences, 47-400 Racibórz, Poland; malgorzata.kuchta@akademiarac.edu.pl; 4 Department of Immunology, Poznan University of Medical Sciences, 60-806 Poznan, Poland; 5 Lifestyle Medicine Lab, Poznan University of Medical Sciences, 60-806 Poznan, Poland

**Keywords:** emotional well-being, psychosocial health, aging women, sociodemographic factors, IPAQ, WHOQOL

## Abstract

**Introduction**: The postmenopausal period is characterized by significant biological and psychosocial changes that can impact women’s physical activity levels and overall quality of life. Physical activity is recognized as one of the key modifiable factors promoting mental health. Still, its role in shaping the emotional domains of quality of life in postmenopausal women remains insufficiently recognized. The study aimed to assess the relationship between physical activity levels and quality of life in postmenopausal women, with particular emphasis on the emotional domains of quality of life, and to determine the role of selected sociodemographic factors. **Materials and Methods**: The cross-sectional study included 174 postmenopausal women. Physical activity levels were assessed using the International Physical Activity Questionnaire (IPAQ), while quality of life was assessed using the WHOQOL questionnaire. Descriptive statistics, Kruskal-Wallis tests with Dunn-Bonferroni post-hoc analysis, and multiple linear regression were used, taking into account age and BMI. **Results**: Higher levels of physical activity were significantly associated with better emotional well-being, higher energy levels, and a more favourable overall health assessment. Physical activity emerged as the strongest and independent variable statistically associated with the emotional domains of quality of life. Educational level and occupational group differentiated the level of physical activity and emotional well-being. **Conclusions**: Physical activity is an important and independent predictor of emotional quality of life in postmenopausal women. The results emphasize the importance of promoting physical activity as part of mental health prevention in this population.

## 1. Introduction

The perimenopausal and postmenopausal periods are associated with numerous biological, psychological, and social changes that can affect both the level of physical activity and the quality of life of women [1,2]. As recent population studies indicate, physical activity levels and sedentary time remain strongly associated with motivation to change health behaviours, as highlighted in a nationwide Italian survey [3].

Physical activity is recognised as one of the key modifiable factors influencing health in middle and late adulthood, improving physiological functioning, mental well-being, and metabolic profile [4,5,6]. At the same time, menopause can contribute to reduced physical activity, increased body weight, and a deterioration in quality of life.

The importance of physical activity for women’s emotional well-being is confirmed by studies showing that avoiding physical activity is associated with higher levels of anxiety and poorer sleep quality [7,8]. At the same time, the level of health-related knowledge and behaviours, which is strongly linked to education, plays an important role in shaping lifestyle and physical activity [9,10]. In addition, environmental and social factors, such as place of residence or gender, can modify the level of physical activity and its health consequences, especially in older populations [11,12,13,14].

Previous studies suggest that physical activity may alleviate menopausal symptoms and contribute to improved quality of life in women; however, the findings remain inconsistent due to heterogeneous study samples and differences in measurement approaches [15,16]. Moreover, relatively few studies have simultaneously examined physical activity and quality of life using standardized and widely accepted instruments such as the International Physical Activity Questionnaire (IPAQ) and the World Health Organization Quality of Life (WHOQOL) questionnaire [17,18]. This indicates a persistent research gap in the integrated assessment of these two domains. An additional gap concerns the insufficient consideration of basic sociodemographic factors—such as education, place of residence, and marital status—as potential determinants of both physical activity and quality of life in menopausal women. Although these variables are commonly analyzed in population-based studies, their role in menopausal populations remains poorly recognized [19,20]. Most existing research focuses primarily on menopausal symptoms, hormonal changes, or somatic health, whereas multifactorial analyses integrating physical activity, quality of life, and sociodemographic variables within a single statistical model are relatively rare. Furthermore, studies applying advanced analytical approaches that allow for a more precise examination of these relationships are limited. Addressing these gaps constituted the main motivation for conducting the present study.

A novel aspect of the present study is the analysis of the interaction between physical activity level (IPAQ) and age, allowing for a more nuanced assessment of how the association between physical activity and emotional domains of quality of life varies across the postmenopausal lifespan.

The aim of the study was to assess the relationship between physical activity levels and quality of life in postmenopausal women, with particular emphasis on the emotional domains of quality of life. An additional aim was to determine the role of selected sociodemographic factors in differentiating physical activity levels and quality of life in the study population. Therefore, the following research questions were posed:

1. Is the level of physical activity of postmenopausal women related to the emotional domains of quality of life (emotional well-being, energy level, overall health assessment)?
2. Does the level of physical activity differ depending on sociodemographic factors such as education, occupational group, marital status, and place of residence?
3. Is physical activity an independent predictor of emotional domains of quality of life after taking into account age, BMI, and age at menopause?
4. What is the relative strength of the impact of physical activity compared to biological and sociodemographic factors on the quality of life of postmenopausal women?

## 2. Materials and Methods

### 2.1. Study Group

Initially, 200 women at different stages of the menopausal transition were recruited. For the purposes of the present study, only women who met the criteria for postmenopause (defined as ≥3 years since the last menstrual period) were included in the final analyses; women in peri- and early perimenopausal stages were excluded.

Ultimately, 174 postmenopausal women were included in the analysis. The study group was characterized using sociodemographic variables, including age, marital status, level of education, place of residence, and occupational category. Body mass index (BMI) was calculated and treated as a biological covariate rather than a sociodemographic variable (Table 1).

The required sample size for comparative analyses was estimated using GPower 3.1, assuming a one-way analysis of variance (ANOVA, fixed effects) as the parametric equivalent of the Kruskal–Wallis test. Assuming a moderate effect size (f = 0.25), a significance level of α = 0.05, a statistical power of 0.80, and three comparison groups, the minimum required sample size was 159 participants. The final sample size in this study (*N* = 174) met these requirements, ensuring adequate statistical power.

Participation in the study was voluntary and anonymous. All participants were informed about the purpose of the study and the possibility of withdrawing at any stage. The study was conducted in accordance with the principles of the Declaration of Helsinki, and approved on 10 December 2020, by the Scientific Research’s Ethic Committee of Jan Dlugosz University in Czestochowa (Resolution Nr KE-U/6/2020).

### 2.2. Research Tools

Physical activity levels were assessed using the International Physical Activity Questionnaire (IPAQ). Based on participants’ responses, total energy expenditure was calculated as MET·min/week and analyzed as a continuous variable, with higher values indicating higher levels of physical activity [17].

Quality of life was assessed using the World Health Organization Quality of Life (WHOQOL) questionnaire, which evaluates multiple domains, including emotional well-being, energy/fatigue, general health, physical functioning, physical and emotional role limitations, social functioning, and pain. Domain scores were expressed as percentage values, with higher scores indicating better perceived quality of life [18].

The data used in this study have been deposited in an open repository and are available at (accessed on 30 December 2025 https://repod.icm.edu.pl/dataverseuser.xhtml?selectTab=notifications).

### 2.3. Statistical Analysis

The normality of variable distributions was assessed using the Shapiro–Wilk test. Due to significant deviations from normality, descriptive data are presented as medians and quartiles (Q1–Q3), as well as interquartile range (IQR) and range (min–max).

Intergroup differences between categories of education level, occupational group, marital status, and place of residence were assessed using the Kruskal–Wallis test. In the case of significant results, Dunn’s post hoc test with Bonferroni correction was applied. Effect sizes for the Kruskal–Wallis test were calculated using the epsilon squared (ε^2^) coefficient. Only complete questionnaires were included in the analyses; therefore, no imputation of missing data was required.

Associations between physical activity and quality of life were examined using multiple linear regression models constructed according to the research questions. In the first set of models, physical activity level (IPAQ MET·min/week) served as the dependent variable, with sociodemographic factors entered as predictors. In the second set of models, WHOQOL quality-of-life domains were specified as dependent variables, while physical activity level, age, body mass index (BMI), and age at menopause were included as predictors. Regression results are reported as standardized coefficients (β), 95% confidence intervals, and coefficients of determination (R^2^ and adjusted R^2^).

An interaction term between physical activity (IPAQ) and age (IPAQ × age) was additionally included to examine whether the association between physical activity and emotional quality-of-life outcomes differs across the postmenopausal age range. This interaction was theoretically justified by age-related changes in physiological reserve, prevalence of chronic conditions, and functional limitations, which may modify the strength of associations between lifestyle behaviors and subjective health outcomes. The interaction analysis was exploratory but conceptually grounded, aiming to provide a more nuanced interpretation of the observed associations.

Total physical activity expressed as MET·min/week was analyzed as a continuous variable, as this approach preserves the full variability of physical activity levels and increases statistical power compared to categorical classifications. This method is commonly applied in population-based studies using the IPAQ questionnaire.

All statistical analyses were performed using the Python (version 3.11; Python Software Foundation, Wilmington, DE, USA) environment (pandas, scipy, statsmodels). The level of statistical significance was set at *p* < 0.05, and the confidence level at 95%.

## 3. Results

Depending on the analysis, physical activity level (IPAQ MET·min/week) was treated either as a dependent variable (in models examining sociodemographic determinants of physical activity) or as an independent predictor (in models explaining quality-of-life outcomes).

Table 2 presents descriptive statistics for variables obtained from the IPAQ and WHOQOL questionnaires. The median total physical activity was 972 MET·min/week (Q1–Q3: 540–1608). In terms of quality of life, the highest median values were recorded for the domains of physical functioning (90 points) and physical role limitations (100 points), whereas the lowest values were observed for overall health assessment (42.5 points) and energy level (52 points).

Subsequent analyses focused on intergroup differences in physical activity levels and emotional domains of quality of life across selected sociodemographic categories. Significant differences were observed between educational levels and occupational categories in both physical activity levels and emotional well-being scores.

Statistically significant intergroup differences in physical activity levels (IPAQ) and emotional domains of quality of life (WHOQOL) are summarized in Table 3. The Kruskal–Wallis test showed that physical activity levels differed significantly depending on education (H = 13.96, *p* = 0.001, ε^2^ = 0.07). Dunn–Bonferroni post-hoc analysis revealed that women with higher education had significantly higher levels of physical activity compared to women with primary education.

Significant differences in physical activity levels were also noted across occupational groups (H = 12.64, *p* = 0.027, ε^2^ = 0.05), with higher MET values observed in managerial occupations compared to the service and trade sectors.

No significant differences in physical activity levels were found depending on marital status or place of residence (*p* > 0.05).

Significant differences in emotional well-being were observed depending on marital status (H = 9.84, *p* = 0.020, ε^2^ = 0.04). Post-hoc analysis showed higher values among married women compared to widows.

Place of residence also differentiated emotional well-being (H = 10.31, *p* = 0.035, ε^2^ = 0.04), with higher values recorded among urban residents compared to rural residents.

The strongest differences occurred due to education (H = 14.88, *p* < 0.001, ε^2^ = 0.08). Women with higher education scored significantly higher on emotional well-being than women with primary and secondary education. In addition, occupational group significantly differentiated this domain (H = 15.21, *p* = 0.009, ε^2^ = 0.06), with the highest scores in educational and social professions.

In the domain of energy and fatigue, no significant intergroup differences were found depending on marital status, place of residence or occupational group (*p* > 0.05). Significant differences were found only in terms of education (H = 9.11, *p* = 0.010, ε^2^ = 0.04), where women with higher education had higher energy levels compared to women with primary education.

Multiple regression analysis (Table 4) showed that the level of physical activity (IPAQ) in postmenopausal women was significantly determined by sociodemographic factors. Higher education and managerial occupations were positive and significant predictors of physical activity levels, explaining a total of 12% of the MET variance. Age and BMI showed no significant association with physical activity levels after controlling for other variables.

Physical activity emerged as the strongest and most stable variable statistically associated with the emotional domains of quality of life. Higher MET levels were significantly associated with better emotional well-being, higher energy levels, and better overall health assessment. These models explained 12% to 16% of the variance in dependent variables. In addition, higher BMI was shown to be a significant negative predictor of emotional well-being and overall health rating, while higher age was associated with lower energy levels and poorer overall health rating. Age at menopause showed no independent association with any of the quality-of-life domains analysed.

The three-dimensional interaction model showed that the positive relationship between physical activity and overall health was moderated by age, with a stronger effect observed in younger women aged ≥50 years, and this effect gradually weakened with advancing age.

A three-dimensional analysis using a regression model that considered the interaction between physical activity level and age showed a significant variation in the impact of IPAQ on overall health assessment depending on the age of women ≥ 50 years. Visualisation in the form of a twisted regression plane with a colour gradient indicated a positive relationship between physical activity and the WHO domain—General health across the entire age range analysed. At the same time, a gradual weakening of the plane’s slope with age was observed, suggesting a decreasing effect of physical activity in older women.

**Figure 1:**
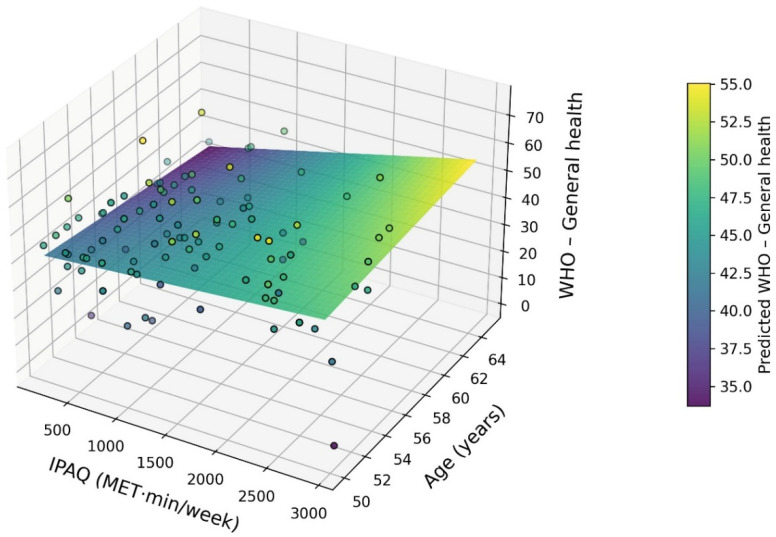
Three-dimensional interaction surface illustrating the moderating effect of age on the relationship between physical activity (IPAQ) and overall health (WHO) in women aged 50 years and older. The colour gradient represents the predicted WHO scores obtained from a multiple regression model with the interaction term IPAQ × age.

Figure 1 presents a three-dimensional interaction surface illustrating the moderating effect of age on the relationship between physical activity (IPAQ) and overall health (WHO) in women aged ≥50 years. The colour gradient further indicates that the highest predicted levels of overall health occur among relatively younger women within this age group who engage in higher levels of physical activity, whereas the lowest values are observed among older women with low MET levels. These findings underscore the significant moderating role of age in the association between physical activity and quality of life.

## 4. Discussion

The aim of this study was to assess differences in physical activity levels and emotional domains of quality of life among postmenopausal women, taking into account selected sociodemographic factors.

Although emotional well-being, energy/fatigue, and general health are conceptually related, they represent distinct WHOQOL domains capturing different aspects of subjective health. Emotional well-being reflects affective states and mood, energy/fatigue captures perceived vitality and physical exhaustion, while general health represents a global self-assessment integrating physical and psychological perceptions. Treating these domains as separate outcomes allows for a more precise interpretation of how physical activity is associated with specific components of quality of life.

The results indicate that physical activity is significantly associated with emotional domains of quality of life in postmenopausal women.

Previous studies focusing specifically on postmenopausal women have shown that regular physical activity is associated with improved emotional well-being and reduced menopausal symptoms [6,21]. Our findings extend this evidence by demonstrating the role of physical activity as an independent correlate of emotional quality of life after accounting for sociodemographic and biological factors.

The results further indicate that educational attainment and occupational status play a significant role in differentiating both the level of physical activity and emotional well-being among the women surveyed.

Women with higher education were characterised by significantly higher physical activity, better emotional well-being, and higher energy levels. These findings are consistent with previous studies reporting positive associations between physical activity and emotional well-being in midlife and older women, while extending existing evidence by demonstrating an age-dependent moderation effect [22]. This pattern may be explained by greater health awareness, better access to health-promoting resources, and more favourable working and living conditions. According to the literature, higher levels of education are associated with more frequent physical activity and better emotional functioning, which may reflect greater control over one’s own health and access to resources that promote healthy behaviours [23].

The occupational group also proved to be an important factor differentiating the analysed variables. The higher level of physical activity observed among women in managerial positions may be related to greater decision-making autonomy, flexible working hours and financial stability. In turn, the higher emotional well-being observed in educational and social professions may reflect a greater sense of meaning in work and stronger social support. According to the occupational stress model, greater autonomy and control over work promote healthy behaviours, including physical activity [24], while educational and social professions are more often associated with higher levels of engagement and emotional well-being [25].

Marital status and place of residence primarily differentiated the emotional components of quality of life, which emphasises the importance of social relationships and environmental context for the mental well-being of postmenopausal women. The importance of interpersonal support and social relationships for emotional quality of life is also confirmed by the results of population studies and meta-analyses [26]. At the same time, the lack of significant differences in the domain of energy and fatigue for most of the socio-demographic factors analysed suggests that this variable is more closely related to biological factors, such as age and menopausal status, than to social conditions.

The youngest premenopausal women, who were excluded from the main analyses, were characterised by higher levels of physical activity and a more favourable profile of emotional domains of quality of life compared to numerically comparable groups of postmenopausal women. Higher levels of physical activity and a more favourable profile of emotional domains of quality of life in premenopausal women were also observed in cohort studies of middle-aged women [27].

Within the postmenopausal population, a gradual decline in physical activity, energy and overall health assessment was observed with age, which further justified the homogeneous selection of the sample for the main analyses. The deterioration in energy levels and subjective health assessment with age and menopausal status is consistent with observations indicating an increase in biological stress during this period of life [1].

The use of a regression model with IPAQ × age interaction and its visualisation in the form of a twisted 3D plane provided an in-depth insight into the mechanism of the relationship between physical activity and the overall health assessment of women over 50. These results indicate that although physical activity remains an important factor contributing to a better quality of life, its relative impact weakens with advancing age. The moderating role of age may be related to the severity of biological changes, the co-occurrence of chronic diseases, and functional limitations that reduce the body’s adaptive potential in older age. Nevertheless, the persistent positive direction of the relationship suggests that physical activity retains its clinical significance even in older age groups. The interaction analysis suggests that although physical activity remains positively associated with overall health across the entire postmenopausal age range, its relative impact decreases with advancing age. From a practical perspective, this indicates that promoting physical activity at earlier stages of postmenopause may yield greater benefits for subjective health and emotional well-being, while in older women physical activity should be combined with other supportive interventions. The literature emphasises that with age, the relative impact of physical activity on health may weaken due to functional limitations and the co-occurrence of chronic diseases [28].

Regression analysis indicated a strong association between physical activity and emotional components of quality of life in postmenopausal women. The particularly strong relationship between the level of physical activity and emotional well-being indicates that regular activity may be an important protective factor in the postmenopausal period, when the risk of low mood, fatigue, and a deterioration in subjective health assessment increases. At the same time, the lack of a significant impact of the age of menopause suggests that the current lifestyle is more important for women’s well-being than the moment of menopause itself. Regular physical activity is recognised as an important protective factor for emotional well-being and quality of life in older age [22].

The negative impact of BMI and age on selected domains of quality of life highlights the need for a holistic approach to the health of postmenopausal women, considering both the promotion of physical activity and weight control and the biological processes of ageing. Literature reports indicate that interventions combining increased physical activity with measures aimed at maintaining a healthy body weight can effectively improve the quality of life of postmenopausal women [29].

The results obtained support the concept that intervention programmes aimed at increasing physical activity levels are an important element in improving the quality of life of postmenopausal women, particularly in emotional domains and subjective health assessment.

### 4.1. Practical Applications

The results obtained indicate that increasing physical activity should be a key element of preventive healthcare and care for postmenopausal women, especially in the context of improving emotional well-being and subjective quality of life.

Regular physical activity may represent a potentially important non-pharmacological strategy that is consistently associated with better emotional well-being in the postmenopausal period, as indicated by previous observational and intervention studies [6,21].

From a clinical practice perspective, it seems reasonable to routinely assess the level of physical activity in postmenopausal women, including the use of standardised tools such as the IPAQ questionnaire [17], and to incorporate the results into individual health recommendations. Particular attention should be paid to women with lower levels of education and those in less autonomous occupations, who, as population studies indicate, are more likely to have low levels of physical activity and poorer health outcomes [23].

Intervention programmes targeting postmenopausal women should focus not only on somatic aspects but also on the emotional components of quality of life. Moderate-intensity physical interventions, tailored to age and health status, can effectively increase energy levels, reduce fatigue and improve mental well-being [30]. From a public health perspective, the results of this study highlight the need to design and implement programmes promoting physical activity both in the workplace and in local communities, with a particular focus on postmenopausal women, which is in line with the current recommendations of the World Health Organisation [31]. Such measures can contribute to improving quality of life, reducing healthcare costs and increasing the social activity of this population.

### 4.2. Research Limitations and Future Studies

This study has several limitations that should be taken into account when interpreting the results. First, the cross-sectional nature of the study does not allow conclusions to be drawn regarding causal relationships between physical activity levels and quality of life. The observed associations may be bidirectional, and their direction should be verified in longitudinal studies.

Physical activity was assessed using the IPAQ questionnaire, which carries the risk of self-report bias and potential overestimation of actual activity levels. Despite the widespread use of IPAQ in population-based studies, the application of objective measurement methods (e.g., accelerometers) could increase the accuracy of physical activity assessment.

Another limitation is the lack of information on the intensity and structure of physical activity, which precludes the assessment of the effects of specific forms of exercise on individual quality-of-life domains. The analyses also did not account for detailed health-related factors, such as hormone replacement therapy, medication use, coexisting chronic diseases, or prior mental health conditions, which may confound the observed associations between physical activity and quality of life. Unmeasured factors such as chronic disease burden, hormone therapy use, or baseline mental health status could potentially attenuate or inflate the observed associations; for example, healthier women may be both more physically active and report better quality of life, which could partially overestimate the strength of the associations. Therefore, the reported relationships should be interpreted with caution.

Future studies should adopt longitudinal and interventional designs to verify the directionality of the observed associations and to distinguish between physical activity domains and intensities, as different types and levels of physical activity—particularly moderate and vigorous exercise—may differentially affect emotional and psychological outcomes in postmenopausal women and help identify the most effective exercise strategies for improving emotional quality of life.

The study was limited to postmenopausal women, which increases sample homogeneity but limits the generalizability of the findings to premenopausal women and male populations. Additionally, the use of purposive sampling may be associated with a risk of selection bias.

Despite these limitations, the homogeneity of the study group, the application of appropriate non-parametric statistical methods, and the consistent reporting of effect sizes enhance the reliability of the obtained results.

## 5. Conclusions

Physical activity is consistently and independently associated with the quality of life of postmenopausal women, particularly in the emotional dimension.

Higher levels of physical activity were consistently associated with better emotional well-being, higher energy levels and a more favourable subjective assessment of health, regardless of age, BMI and age at menopause.

Sociodemographic factors, such as education and occupational status, mainly differentiated the level of physical activity, indicating their indirect role in shaping quality of life. At the same time, the lack of a significant impact of menopausal age emphasises the importance of current health behaviours, rather than the timing of menopause itself, for the well-being of postmenopausal women.

Health promotion programmes aimed at increasing physical activity may contribute to improvements in emotional quality of life among postmenopausal women, particularly in the area of mental health. Further research, especially longitudinal and interventional studies, is needed to clarify causal relationships and to identify the optimal forms and intensity of physical activity in this population.

## Figures and Tables

**Table 1:** Characteristics of the study participants.

Variable	Value
Inclusion criteria	Age ≥ 18 years; declared menopause; complete questionnaire data
Age (years)	54 (IQR = 8; range: 45–78)
Height (cm)	165 (IQR = 7; range: 153–178)
Body weight (kg)	68 (IQR = 16.4; range: 42–108)
Menopausal status	
– Postmenopausal (>3 years since last menstruation)	46%
– Perimenopausal (1–3 years without menstruation)	39%
– Early perimenopause (<12 months without menstruation)	17%
**Childbirth history**	
– At least one childbirth	97%
– No childbirth	2 participants
**Marital status**	
– Married	69.5%
**Education level**	
– Higher education	54.1%
– Secondary education	33.3%
Occupational category	
– Medical professions	43.0%
– Education sector	24.5%
– Retired	smallest group
Place of residence	Various regions of the country

Data on age, height, and body weight were obtained through self-report in the demographic questionnaire. Values are presented as median (IQR; range) or percentage. Bold type indicates sociodemographic characteristics of the participants.

**Table 2:** Descriptive statistics of selected variables.

Questionary	Variable	Median	Q1–Q3	IQR	Min–Max
IPAQ	MET (min·MET/tydz.)	972.0	540.0–1608.0	1068.0	120–8920
WHOQOL	Physical functioning	90.0	75.0–100.0	25.0	20–100
	Role limitation (physical)	100.0	50.0–100.0	50.0	0–100
	Role limitation (emotional)	83.3	33.3–100.0	66.7	0–100
	Energy/Fatigue	52.0	45.0–58.0	13.0	20–80
	Emotional well-being	52.0	46.0–64.0	18.0	24–88
	Social functioning	75.0	50.0–87.5	37.5	0–100
	Pain	55.0	45.0–75.0	30.0	10–100
	General health	42.5	35.0–50.0	15.0	15–80

**Table 3:** Intergroup differences in physical activity levels (IPAQ) and emotional domains of quality of life (WHOQOL) according to selected sociodemographic factors. Groups compared: education level, occupational category, marital status, place of residence.

Factor	Variable	H	*p*	ε^2^	Post-Hoc (Dunn–Bonf)
Marital status	Emotional well-being	9.84	0.02	0.04	married woman > widow
Place of residence	Emotional well-being	10.31	0.035	0.04	city > countryside
Education	IPAQ (MET)	13.96	0.001	0.07	higher > primary
Education	Emotional well-being	14.88	<0.001	0.08	higher > primary/secondary
Occupational group	IPAQ (MET)	12.64	0.027	0.05	management > services
Occupational group	Emotional well-being	15.21	0.009	0.06	education > services

IPAQ—International Physical Activity Questionnaire (MET·min/week); WHOQOL—World Health Organization Quality of Life questionnaire; H—Kruskal–Wallis test statistic; *p*—level of statistical significance; ε^2^—epsilon squared effect size; Dunn–Bonf—Dunn post-hoc test with Bonferroni correction.

**Table 4:** Multiple regression—only statistically significant predictors (*p* < 0.05).

Significant Predictor	Dependent Variable	β*	95% CI	*p*	Adj, R^2^
Education (higher)	IPAQ (MET)	0.21	61.2; 563.8	0.016	0.12
Occupational group (management)		0.18	22.7; 553.1	0.034	
IPAQ (MET)	WHO—Emotional well-being	0.32	0.0021; 0.0061	<0.001	0.16
BMI		−0.15	−0.48; −0.01	0.048	
IPAQ (MET)	WHO—Energy/Fatigue	0.24	0.0009; 0.0051	0.006	0.12
Age		−0.19	−0.38; −0.04	0.012	
IPAQ (MET)	WHO—General health	0.23	0.0011; 0.0047	0.002	0.13
Age		−0.14	−0.35; −0.01	0.041	
BMI		−0.15	−0.57; −0.02	0.038	

IPAQ—International Physical Activity Questionnaire (MET·min/week). β*—standardised regression coefficient. CI—95% confidence interval.

## Data Availability

The data used in this study have been deposited in an open repository and are available at (accessed on 30 December 2025 https://repod.icm.edu.pl/dataverseuser.xhtml?selectTab=notifications).
